# Minor Myeloid Leukemic Populations in Pediatric Acute Lymphoblastic Leukemia: A Potentially Underrecognized Mixed-Phenotype Tumor?

**DOI:** 10.3390/ijms27198816

**Published:** 2026-10-02

**Authors:** Alexandra Semchenkova, Polina Lavrova, Vladimir Kozeev, Irina Demina, Ekaterina Mikhailova, Julia Roumiantseva, Svetlana Lagoyko, Anna Kazakova, Galina Novichkova, Alexander Karachunskiy, Elena Zerkalenkova, Alexander Popov

**Affiliations:** Dmitry Rogachev National Medical Research Center of Pediatric Hematology, Oncology and Immunology, 1 Samory Mashela St., Moscow 117198, Russia

**Keywords:** acute lymphoblastic leukemia, mixed-phenotype leukemia, lineage switch

## Abstract

In “bilineal” mixed-phenotype acute leukemia (MPAL), the proportions of lymphoid and myeloid leukemic cells may differ substantially. A myeloid component may constitute <10% of bone marrow (BM) cells and show subtle immunophenotypic abnormalities detectable by multicolor flow cytometry (MFC). The significance of minor myeloid leukemic populations in otherwise acute lymphoblastic leukemia (ALL), including their association with lineage switch, remains uncertain. We retrospectively reviewed diagnostic MFC data from 5094 children with B-cell precursor or T-cell ALL. An additional minor myeloid leukemic population (<10% of BM) was identified in 92 ALL cases (1.8%), with a median of 3.0% of BM cells (0.2–9.2%). The frequency of ALL cases harboring a minor myeloid leukemic population was comparable to that of overt “bilineal” MPAL (with both populations ≥ 10% of BM cells), diagnosed during the same period. The myeloid populations expressed CD33, CD64, CD14, lysozyme, and markers shared with the dominant lymphoblastic population (CD19/CD7, CD34, CD117). Common genetic findings were high hyperdiploidy (*n* = 14), *KMT2A* rearrangements (*n* = 13), and *BCR*::*ABL1* (*n* = 8). Although no expansion was documented in most patients, 6 of 83 newly diagnosed patients (7.2%) developed lineage switch to acute myeloid leukemia or overt “bilineal” MPAL. Most cases were managed successfully with standard ALL-directed therapy.

## 1. Introduction

Acute lymphoblastic leukemia (ALL), the most common pediatric cancer, arises from malignant transformation of lymphoid precursors committed to the B- or T-cell lineage and results in pathological displacement of normal hematopoiesis [1]. Accurate lineage assignment is essential for therapeutic stratification and relies predominantly on multicolor flow cytometry (MFC) [2].

MFC is indispensable for characterizing rare and biologically ambiguous leukemia subtypes, particularly mixed-phenotype acute leukemia (MPAL). This diagnostically challenging entity represents approximately 2–5% of acute leukemia cases and is associated with an inferior prognosis compared to lineage-restricted ALL or acute myeloid leukemia (AML) [3]. Diagnostic recommendations for MPAL are outlined in the 2022 WHO classification [4]. Difficulties typically arise in MPAL cases with a single cell population that co-expresses antigens of different lineages. In some cases, however, MPAL presents as two immunophenotypically distinct blast populations of different lineages—a pattern previously termed “bilineal” leukemia. One leukemic population may substantially predominate over another that may lack prominent antigen aberrancies. Notably, the WHO criteria stipulate that the total leukemic burden must comprise ≥20% of total bone marrow (BM) cells—the general threshold for leukemia diagnosis—whereas the relative contributions of individual populations in “bilineal” MPAL remain unspecified.

This lack of a defined lower threshold for each leukemic population has practical diagnostic consequences. In routine practice, small subpopulations belonging to a lineage distinct from the dominant blast population are often not systematically identified or reported, particularly when their distinction from normal counterparts depends on subtle immunophenotypic deviations. Virtually no data exist on whether the presence of such small subpopulations (often myeloid or monocytic) truly reflects MPAL. It is likewise unknown whether these cells foreshadow subsequent lineage switch (LS)—a well-recognized but poorly predictable event in acute leukemia [5,6]—or instead remain clinically silent.

At the same time, the immunophenotypic characteristics of these minor myeloid populations and the reliability with which they can be detected by routine diagnostic MFC remain poorly defined. Therefore, the present study aimed to identify and characterize minor myeloid leukemic populations (<10% of BM cells) in pediatric ALL and to describe the clinical course of affected patients, with particular attention to subsequent LS events.

## 2. Results

The study cohort included 5094 cases of ALL processed at our institution between August 2019 and September 2024: 4420 cases of B-cell precursor ALL (BCP-ALL) and 674 cases of T-cell ALL (T-ALL). In 92 cases (1.8%), MFC revealed a predominant lymphoblastic population accompanied by a minor subset of leukemic cells with myeloid differentiation (<10% of total BM cells) exhibiting subtle immunophenotypic deviations from normal myeloid counterparts. This group comprised 53 boys and 39 girls, and the median age at diagnosis was 8.8 years (range 5 days–18.0 years). Eight patients were younger than 1 year. The analyzed MFC data were obtained predominantly at initial diagnosis (*n* = 83; 90.2%), with relapse cases comprising 9.8% (*n* = 9). According to MFC, 74 cases were identified as BCP-ALL (1.7% of overall BCP-ALL cases) and 18 cases as T-ALL (2.7% of overall T-ALL cases).

The study selection process and the availability of laboratory data are summarized in Figure 1. Among the 92 patients included in the final cohort, complete laboratory data, including BM morphology, myeloperoxidase (MPO) cytochemistry, and cytogenetics, were available for 64 patients (69.6%). Additional molecular investigations, including RNA sequencing (RNAseq) and whole-genome sequencing (WGS), were available for 34 patients. The availability of diagnostic data varied across patients, primarily for logistical reasons and, in some cases, inadequate specimen quality or insufficient residual biological material.

### 2.1. Morphology and Cytochemistry

Morphological assessment was available for 71 out of 92 patients (77.2%). The median BM blast percentage was 86.2% (range 28.0–99.6%). Blast morphology was consistent with ALL in 53 cases (74.6%), with L2 morphology being the most frequent (*n* = 32). AML was suspected morphologically in one case (M5a FAB-subtype). In 5 cases, bilineal leukemia was suspected. Blast lineage remained unspecified by morphology/cytochemistry in 12 cases (Figure 2A).

A morphologically homogeneous blast population was observed in most cases (*n* = 63; 88.7%). Significant blast heterogeneity, primarily in cell size and cytoplasm-to-nucleus ratio, was identified in eight cases (11.3%) (Figure 2B). Auer rods, pathognomonic of myeloid differentiation, were detected in two cases (2.8%); in one of these, they were present only in a small subset of blasts.

MPO and Sudan Black B (SBB) staining results were available for 67 patients (Figure 2C,D). MPO staining was negative in most cases (*n* = 45, 67.2%). Positivity in ≥3% of blasts—a threshold indicative of myeloid differentiation—was observed in 10 cases (range 3–8%), whereas 12 cases showed weak reactivity in only 1–2% of blasts. Similarly, SBB staining was strongly positive (≥3%) in 16 cases (23.9%), negative in 41 cases (61.2%), and weak (<3%) in 10 cases (14.9%).

Periodic acid–Schiff (PAS) reaction results were available in 64 patients (Figure 2E). PAS negativity was observed in 9 cases (14.1%). In 20 samples (31.3%), the majority of blasts were PAS-positive. A granular or block-like pattern was observed in 53 cases (82.8%), while a diffuse granular pattern was seen in only 2 cases (3.2%). α-Naphthyl acetate esterase (NSE) activity was positive in 31 of 63 evaluated patients (Figure 2F).

### 2.2. Immunophenotyping

In all 92 samples, MFC revealed a dominant lymphoblastic population with a median representation of 81.3% (range 15.7–97.0%) of total BM cells. The coexisting myeloid leukemic subset was present at a median proportion of 3.0% (range 0.2–9.2%) of total BM cells.

Of 92 cases, 74 were diagnosed as BCP-ALL and 18 as T-ALL. According to the European Group for the Immunological Characterization of Leukemias (EGIL) criteria, the BII immunophenotype was most prevalent among BCP-ALL cases (*n* = 54), followed by BI (*n* = 18) and BIII (*n* = 2). Among T-ALL cases, TII was observed in 17 patients (10 cases of early T-cell precursor (ETP-) ALL and 7 non-ETP-ALL cases), and one patient had TIII immunophenotype (Figure 3). At least one myeloid antigen was co-expressed by the dominant lymphoblastic population in 62 of 92 patients (67.4%). The most common myeloid co-expressions in lymphoblastic populations were CD33 (*n* = 39) and CD15 (*n* = 19).

The minor myeloid leukemic populations predominantly expressed CD33 (*n* = 88), CD64 (*n* = 79), lysozyme (*n* = 77), CD14 (*n* = 70), and CD15 (*n* = 44) (Table 1). This combination of markers closely mirrors the phenotype of normal monocytes, indicating monocytic lineage commitment in the majority of cases. In addition to this cell population, residual phenotypically normal monocytes without any antigen aberrations were observed in nearly half of the cases (*n* = 38; range 0.3–3.5%).

The minor myeloid leukemic cells expressed MPO in 38 of 92 cases (41.3%) at a median level of 87% (range 28.0–100.0%). MFC identified MPO expression in all MPO-positive cases by cytochemistry (*n* = 10). In the remaining MPO-positive cases by MFC, cytochemistry revealed blasts negative for MPO in eight patients and blasts with MPO positivity below 3% in eight patients. Cytochemical data were unavailable for 12 patients who were MPO-positive by MFC.

In contrast to normal mature monocytes (positive for lysozyme, CD14, CD33, CD64 and negative for immaturity-associated CD34, CD117), the myeloid leukemic cells expressed leukemia-associated markers—CD19 in 67 of 74 BCP-ALL cases, CD7 in 13 of 18 T-ALL, CD34 in 54 of 92 ALL cases—supporting their leukemic nature and suggesting a relationship to the dominant lymphoblastic population. In all samples, these cells were strongly CD45-positive, irrespective of CD45 expression level in the dominant lymphoblastic population. Antigen expression details are summarized in Table 1.

Of interest, among nine relapsed patients in our cohort, initial diagnostic MFC data were available for eight. Their review revealed the same minor myeloid leukemic population in five cases.

Representative antigen expression patterns of lymphoblasts and minor myeloid leukemic population are shown in Figure 4.

### 2.3. Cytogenetics and Molecular Genetics

Cytogenetic analysis was performed in 88 of 92 patients (95.7%) (Figure 5). In three of these 88 cases, conventional karyotyping failed due to insufficient metaphase spreads, and no risk-stratifying rearrangements were detected by fluorescence in situ hybridization (FISH). Among the remaining informative cases (*n* = 85), the most prevalent abnormalities were high hyperdiploidy (*n* = 14) and *KMT2A* rearrangements (*n* = 13). The latter is commonly associated with increased lineage plasticity and MPAL [7,8]. The t(9;22)(q34;q11)/*BCR*::*ABL1* fusion was identified in eight cases, comprising two p210-positive and six p190-positive cases. *ZNF384* rearrangements, another genetic lesion associated with MPAL [8,9], were identified in six patients, all involved in t(12;19)(p13;p13)/*TCF3*::*ZNF384*. *BCL11B* rearrangements were found in three patients. Collectively, MPAL-associated genetic lesions accounted for only 30 of 85 informative cases (35.3%).

Isolated chromosomal gains or losses (monosomy 7, trisomy 8, etc.) were observed in 11 cases, and a normal karyotype without risk-stratifying rearrangements was found in six patients. Individual patients also harbored rearrangements of *CRLF2*, *DUX4*, *IKZF1*, *PDGFRB*, and *RUNX1*, as well as intracromosomal amplification of chromosome 21 (iAMP21).

Additional molecular studies identified *FLT3*-ITD in two ETP-ALL cases, a somatic *TP53* mutation in one BCP-ALL case, and *PAX5* P80R in two BCP-ALL cases.

A summary of cytogenetic and molecular findings is presented in Table A1 (Appendix A).

### 2.4. Flow Cell Sorting

In 5 of the 92 patients, the lymphoblastic and minor myeloid leukemic populations were isolated by flow cell sorting to assess their clonal relationship using cytogenetic or molecular methods. In four patients, leukemia-associated chromosomal abnormalities were initially identified in unsorted BM samples by conventional cytogenetic analysis. The sorted populations were subsequently analyzed by FISH, which detected the same chromosomal abnormality in both populations in all four cases. In the remaining patient, conventional cytogenetic analysis of the unsorted BM sample showed a normal male karyotype (46,XY). The lymphoblastic and minor myeloid leukemic populations were therefore sorted separately and analyzed for immunoglobulin gene rearrangements by multiplex PCR. An identical clonal immunoglobulin heavy-chain (*IGH*) rearrangement was detected in both populations. These findings support a shared clonal origin of the lymphoblastic and minor myeloid populations in all five evaluated cases. The results are summarized in Table 2.

### 2.5. Treatment and Outcomes

Among newly diagnosed patients (*n* = 83), most received therapy according to the contemporary national ALL-MB 2015 protocol (*n* = 66). Smaller subsets of patients were treated with BFM-based protocols (*n* = 4), the ALL-MB 2019 Pilot (*n* = 1), ALL-2016 (*n* = 1), and the infant-specific protocols ALL-Baby 2021 (*n* = 4) and MLL-Baby (*n* = 3). One patient received induction therapy according to the EsPh-ALL BFM protocol, followed by ALL-MB-2015 consolidation. Two patients were switched from ALL-directed to AML-type therapy: one due to an initial morphological classification as AML (with an ETP-ALL immunophenotype) and a poor response on day 15 of induction, and the other due to expansion of the myeloid leukemic population by the end of induction (EOI). One patient died before therapy initiation. Risk groups were known for 71 patients: according to protocol-specific stratification criteria, 16 patients were assigned to the standard-risk groups, 23 to the intermediate-risk groups, and 32 to the high-risk groups. Overall, seven patients were lost to follow-up after diagnosis and three after EOI.

Among patients studied at relapse (*n* = 9), first-line therapy had been administered according to ALL-MB (*n* = 8) or the ALLTogether protocol (*n* = 1). Following relapse, patients were treated according to ALL-MB REZ 2014/2016 (*n* = 6) and ALL-BFM REZ 2002/2013 (*n* = 2); one patient died before salvage therapy initiation.

Among patients with available follow-up data (*n* = 80), eight were refractory to therapy and five experienced disease relapse; of these, one patient was experiencing a second relapse, and two were experiencing a third relapse. In addition to conventional chemotherapy, blinatumomab was administered to 25 patients, CD19-targeted CAR-T cell therapy to six, and inotuzumab ozogamicin to four; more than one type of immunotherapy was used in six cases. Seventeen patients underwent allogeneic hematopoietic stem cell transplantation (HSCT). Treatment details are represented in Table 3.

Follow-up MFC assessments were performed according to the schedules of the treatment protocols applied. Patients with a minor myeloid leukemic population were not subjected to additional or more intensive MFC monitoring solely on the basis of this finding.

### 2.6. Lineage Switch

LS was defined as a change in the dominant blast lineage confirmed by immunophenotyping, with preservation of the original genetic lesion when assessable. LS was documented in 6 of 83 patients (7.2%) evaluated at initial diagnosis. Three patients with BCP-ALL (two BI and one BII) and one patient with ETP-ALL switched to AML, whereas two patients with BCP-ALL (one BI and one BIII) developed “bilineal” MPAL with a predominant myeloid component. All six patients initially received ALL-directed therapy. Of note, among the nine patients who entered the study at the time of relapse, no subsequent LS events were documented.

Five LS events occurred by day 15 of induction. The corresponding induction regimens included dexamethasone, vincristine, daunorubicin, PEG-asparaginase, and triple intrathecal therapy with methotrexate, cytarabine, and prednisolone. In these five patients, the lineage-switched population remained detectable for a median of 125 days (range 25–148 days). The remaining LS event was documented at relapse, 10 months after the initial diagnosis, following ALL-directed chemotherapy based on the same core agents and subsequent treatment with blinatumomab.

Among the five early LS events, four occurred in patients with *KMT2A*-rearranged BCP-ALL and one in a patient with ETP-ALL without specific genetic abnormalities. The BCP-ALL patient who switched at relapse had t(4;14)(q35;q32)/*IGH*::*DUX4* at initial diagnosis, which was preserved at LS.

In five of six cases, the minor myeloid leukemic population identified at diagnosis became dominant at the time of LS. The exception was the ETP-ALL patient, in whom the dominant population at LS retained the same lymphoblast-like features but had lost the key lineage-defining antigen iCD3. Multiple myeloid co-expressions led to the formal reclassification as myeloid leukemia, though the cells did not constitute a monocytic population. This patient achieved remission on ALL-directed therapy but died 22 months after allogeneic HSCT from an unspecified cause.

Among patients with early LS (*n* = 5), four continued ALL-directed therapy and achieved remission. The remaining patient was switched to AML-directed therapy and also achieved remission. The patient who developed AML at relapse received AML-directed therapy but died from progressive disease.

Among the remaining 77 newly diagnosed patients without documented LS, the median clinical follow-up duration was 44.4 months (range 0.4–82.2 months). Of these, 61 (79.2%) had at least 12 months and 57 (74.0%) had at least 24 months of clinical follow-up.

Of note, in another patient with ETP-ALL who did not meet the criteria for LS, myeloid leukemic cells persisted alongside the lymphoblastic population for one month after diagnosis and disappeared following high-dose chemotherapy blocks. Apart from the six LS cases and this transient expansion, the minor myeloid leukemic populations generally showed no documented expansion during the available follow-up.

The clinical, immunophenotypic, genetic, and therapeutic characteristics of all six LS cases are summarized in Table 4.

## 3. Discussion

The present study provides a systematic characterization of ALL cases in a pediatric cohort, in which a dominant lymphoblastic subpopulation is accompanied by a minor (<10% of BM cells) myeloid leukemic population. Among 5094 patients evaluated over a five-year period, we identified 92 such cases (1.8%), representing 1.7% of overall BCP-ALL cases and 2.7% of overall T-ALL cases. During the same period, 71 cases of overt “bilineal” MPAL (1.4%) were identified at our institution [10], defined by each leukemic population comprising ≥ 10% BM burden. Thus, ALL cases harboring a minor myeloid leukemic population were identified at a frequency comparable to that of overt “bilineal” MPAL, highlighting a potential underestimation of cases with lineage ambiguity.

The interpretation of such cases differs between current classification systems. According to the 2022 WHO guidelines, the total leukemic blast population must constitute at least 20%, with the individual populations classified according to standard AML and ALL criteria [4]. By contrast, the International Consensus Classification (ICC) recommends that a divergent population constitute at least 5% of total cells to support classification as “bilineal” MPAL [11]. When the smaller aberrant population represents less than 5%, the diagnosis is based on the predominant leukemic population, accompanied by a descriptive qualifier, such as “predominantly ALL with a small leukemic population of myeloid lineage detected, of uncertain significance,” with further details provided in the diagnostic report [12]. Thus, no uniform consensus currently exists regarding the minimum proportion required for each lineage-defined population in “bilineal” MPAL.

MFC is a highly sensitive tool for determining blast lineage in acute leukemia and is particularly crucial for MPAL diagnosis. In “bilineal” MPAL, leukemic blasts may appear morphologically immature and lack distinctive cytological features, whereas MFC can resolve them into phenotypically distinct populations [13]. Cytochemical detection of MPO activity or nonspecific esterase activity may support myeloid or monocytic differentiation, yet the sensitivity of these assays for detecting minor subpopulations is inherently limited [13]. The assessment of MPO expression is particularly important in the diagnostic work-up of leukemia of ambiguous lineage [4]. In our cohort, cytochemical MPO positivity meeting the diagnostic threshold was documented in 10 patients, while an additional 12 patients had rare MPO-positive cells below the 3% threshold. By MFC, intracellular MPO expression was detected in the minor myeloid population in 38 patients, involving 28.0–100.0% of cells within that population. Technically, MFC allows population-specific gating and the evaluation of substantially larger numbers of cells, facilitating the detection of MPO expression within a small population. Immature myeloid cells may contain detectable intracellular MPO protein but have insufficient enzymatic activity to produce a strong cytochemical reaction.

Myeloid leukemic cells in our cohort consistently expressed classical monocytic markers, including CD64, CD33, CD14, and lysozyme, while simultaneously retaining leukemia-associated antigens such as CD19 (in BCP-ALL) and CD7 (in T-ALL), as well as CD34 or CD117. This combination of markers supported the abnormality and clonal nature of the minor myeloid populations. We previously reported the isolation of such minor myeloid and lymphoblastic leukemic populations by flow cell sorting. Their shared clonal origin was demonstrated using cytogenetic and molecular analyses [14]. In the present cohort, cell sorting and subsequent genetic or molecular testing were performed at diagnosis in 5 of 92 patients, confirming a shared clonal abnormality in both populations in all five cases. However, such confirmation was not feasible in cases with extremely small aberrant populations.

With regard to overall immunophenotypic features of ALL, our cohort was enriched for BI-ALL (*n* = 18, 24.3% of BCP-ALL cases) and ETP-ALL (*n* = 10, 55.6% of T-ALL cases), compared to unselected pediatric ALL cohorts, in which ETP-ALL typically accounts for only 11–16% of T-ALL cases, and BI-ALL represents 5–15% of BCP-ALL cases [15,16]. According to our data, BI-ALL represented 4.1% (*n* = 180) of overall BCP-ALL cases (*n* = 4420) and ETP-ALL represented 12.3% (*n* = 83) of overall T-ALL cases (*n* = 674) during the study period. The overrepresentation likely reflects a bias toward immunophenotypically more immature subtypes in ALL cases with minor myeloid leukemic subpopulations.

The genetic landscape of our cohort was expectedly enriched for lesions associated with MPAL and lineage plasticity. *KMT2A* rearrangements (*n* = 13), *BCR*::*ABL1* (*n* = 8), *ZNF384* fusions (*n* = 6), and *BCL11B* rearrangements (*n* = 3) together accounted for 35.3% of genetically characterized cases. However, in the majority of cases, no MPAL-associated genetic abnormalities were identified. Instead, the predominant finding was high hyperdiploidy (*n* = 14). Hyperdiploidy is distinctly uncommon in MPAL [17]. Its high frequency in our cohort most likely reflects the underlying epidemiology of BCP-ALL, in which high hyperdiploidy represents the most common cytogenetic subgroup, irrespective of the presence or absence of a myeloid leukemic component [18].

According to the report by A. Kovach et al., monocytic differentiation of blast subpopulations in ALL was associated with *CRLF2* rearrangement: among 223 de novo BCP-ALL cases, 7 of 31 (22.6%) *CRLF2*-rearranged cases demonstrated a partial monocytic component comprising 0.5–7.0% of total leukemic cells [19]. Other studies have shown that *CRLF2* rearrangement is associated with lineage plasticity, enabling transformation from the lymphoid to the myeloid lineage [20]. In our cohort, *CRLF2* rearrangement was detected in two patients, one of whom also harbored iAMP21; neither experienced subsequent lineage conversion.

Lineage switch, defined as the immunophenotypic transformation of the dominant leukemic clone, has been reported in 6–9% of relapsed ALL cases [5]. LS has increasingly been recognized following antigen-directed immunotherapy, particularly CD19-targeted CAR T-cell therapy and blinatumomab, both of which exert selective pressure favoring the outgrowth of antigen-negative clones [21]. However, LS cases following standard chemotherapy alone have also been described [22]. Notably, five of the six LS events in the present work occurred during early induction. Early lineage-divergent changes during induction are not unique to our cohort and have been described in BCP-ALL with specific genetic alterations, including *DUX4* and *ZNF384* rearrangements and *PAX5* P80R mutations [23,24]. Such early changes may be transient and should not automatically be equated with persistent LS. In our cohort, however, the myeloid leukemic populations persisted for a median of 4.1 months after diagnosis, in some cases after the B-lymphoblastic population was no longer detectable. This persistence supports their interpretation as sustained lineage transitions rather than isolated transient phenotypic shifts. Consistent with the known genetic associations of lineage plasticity [23], four of the six patients with LS harbored *KMT2A* rearrangements, and one had an *IGH*::*DUX4* rearrangement.

The transition from ETP-ALL to a myeloid phenotype observed in one patient likely reflects a distinct biological basis for lineage plasticity. ETP-ALL arises from immature thymic progenitors that retain myeloid differentiation potential. Accordingly, reported lineage transitions in ETP-ALL are typically characterized by loss of lineage-defining T-cell markers and acquisition or persistence of a myeloid-associated immunophenotype, leading to reclassification as AML or another acute leukemia category [25]. This pattern differs from that observed in BCP-ALL, in which LS commonly involves expansion of a myeloid/monocytic population accompanied by loss or marked reduction of the original B-lineage component.

Minor myeloid leukemic populations can be detected by MFC in a subset of pediatric ALL cases and may represent diagnostically challenging cases with lineage ambiguity. Although no lineage progression was documented in most patients during the available follow-up, persistent lineage switch occurred in 6 of 83 newly diagnosed cases, and four of them had *KMT2A* rearrangements. The majority of cases were managed successfully with standard ALL-directed therapy. Nevertheless, the retrospective, single-center design, incomplete follow-up in some patients, treatment heterogeneity, and the limited number of lineage-switch events preclude definitive conclusions regarding their prognostic significance or implications for treatment selection.

## 4. Materials and Methods

We conducted a retrospective analysis of diagnostic data from 5094 pediatric patients with BCP-ALL and T-ALL collected between August 2019 and September 2024. Cases of interest were defined by the presence of a minor population of myeloid cells characterized by co-expression of leukemia-associated antigens and subtle immunophenotypic deviations from normal counterparts.

### 4.1. Multicolor Flow Cytometry and Flow Cell Sorting

Diagnostic immunophenotyping was performed according to protocols of the Russian-Belarusian Cooperative Group for Pediatric Acute Leukemia Diagnostics [26]. BM samples were stained with panels of fluorescently labeled monoclonal antibodies as follows:

1. BCP-ALL: CD2, CD3, iCD3 ^1^, CD7, CD10, CD11c, CD13, CD14, CD15, CD19, CD20, CD22, CD24, CD33, CD34, CD38, CD45, CD56, CD58, CD64, CD66b, iCD79a ^1^, CD117, NG2, lambda, kappa, lysozyme, MPO, IgM, iIgM ^1^;
2. T-ALL: CD1a, CD2, CD3, iCD3 ^1^, CD4, CD5, CD7, CD8, CD10, CD11b, CD11c, CD14, CD19, CD33, CD34, CD48, CD56, CD64, CD66b, iCD79a ^1^, CD99, CD117, HLA-DR, TCRαβ, TCRγδ, lysozyme, MPO.

^1^ i—intracellular staining.

Antibody clones and fluorochromes varied throughout the study period depending on reagent availability. Erythrocyte lysis was performed using FACSLyse buffer (Becton Dickinson, BD, Franklin Lakes, NJ, USA). Intracellular staining was performed using fixation/permeabilization kits (IntraSure, BD; IntraPrep, Beckman Coulter, Indianapolis, IN, USA). Data acquisition was performed on Navios (Beckman Coulter) and FACSCanto II (BD) flow cytometers, with a minimum threshold of 50,000 acquired events per sample. Both instruments were operated under standardized laboratory procedures, including routine instrument performance monitoring, fluorescence compensation, and quality-control procedures. Data analysis was performed using Kaluza 2.1 software (Beckman Coulter). Leukemic burden was reported as the percentage of leukemic cells of each lineage relative to total BM cells. Normal hematopoietic cells with light-scatter properties similar to those of malignant populations served as internal negative controls.

Despite occasional changes in antibody clones, the uniform diagnostic criteria were maintained throughout the study period. The dominant lymphoblastic population was subclassified according to its maturation stage using the adapted EGIL criteria [15]. The 2022 WHO criteria were also used for MPAL diagnosis; in samples containing two distinct leukemic populations, each population was characterized separately and independently assigned to a lineage [4]. Myeloid lineage assignment required expression of at least two myeloid/monocytic markers among CD33, CD117, CD13, CD15, CD64, and MPO, in the absence of features definitive of BCP-ALL or T-ALL in that population. Antigen positivity thresholds were set at ≥20% for surface markers and ≥10% for intracellular markers.

A consistent gating strategy was applied to all cases. First, the dominant lymphoblastic population was identified based on CD45 expression, light-scatter characteristics, and lineage-associated antigen profile. The regions corresponding to myeloid progenitors and mature monocytes were then systematically examined for discrete cell populations with coordinated expression of myeloid/monocytic markers and leukemia-associated phenotypic abnormalities. These included expression of markers shared with the dominant lymphoblastic population, such as CD19 in BCP-ALL or CD7 in T-ALL. The immunophenotype of each suspected population was compared with that of residual phenotypically normal monocytes or myeloid progenitors within the same sample, when present. Suspected populations were back-gated and evaluated across all relevant marker combinations to verify their consistent identification and distinction from normal hematopoietic cells.

Flow cell sorting was performed in “Purity” mode using a FACSAria III cell sorter (BD). Suitable combinations of antibodies were determined in each case based on the diagnosed immunophenotype. Sample preparation has been described previously in detail [14]. For FISH analysis, 20,000 cells from each population were sorted in duplicate at diagnosis. For clonality analysis by multiplex PCR, 60,000–100,000 cells from each population were sorted.

### 4.2. Morphology and Cytochemistry Studies

Diagnostic BM aspirate smears were evaluated by Romanowsky–Giemsa staining. Cytochemical analysis included myeloperoxidase (MPO), Sudan Black B (SBB), periodic acid–Schiff (PAS), and α-naphthyl acetate esterase (NSE) with sodium fluoride inhibition. Morphological classification was based on the French–American–British (FAB) criteria [27].

### 4.3. Cytogenetics and Molecular Studies

Conventional karyotyping was performed using G-banding without mitogenic stimulation. Fluorescence in situ hybridization (FISH) for recurrent chromosomal aberrations was carried out according to the manufacturers’ protocols. Karyotypes were described according to the 2024 International System for Human Cytogenomic Nomenclature (ISCN) [28]. All primary BCP-ALL samples were analyzed for *KMT2A* rearrangements, t(9;22)(q34;q11)/*BCR*::*ABL1* and t(12;21)(p13;q22)/*ETV6*::*RUNX1*. In the absence of these abnormalities, additional testing for *TCF3* rearrangements was performed. T-ALL samples were investigated for t(9;22)(q34;q11)/*BCR*::*ABL1* and *TLX3* rearrangements. Other non-risk-stratifying structural abnormalities detected by karyotyping were confirmed by targeted FISH. Where applicable, fusion transcripts were confirmed by RT-PCR. In cases with suspected hyperdiploidy based on karyotyping, centromeric probes for chromosomes 4, 10, and 17 were applied.

In 34 patients, data on additional molecular studies were available, including RNAseq and WGS. In patients with early T-cell precursor ALL (ETP-ALL), *FLT3* mutational status was assessed by fragment analysis and Sanger sequencing, and additional testing for *BCL11B* rearrangements was performed.

Analysis of patient-specific rearrangements in flow-sorted cells by FISH and analysis of clonal *IG/TCR* rearrangements by multiplex PCR were described previously in detail [14].

### 4.4. Statistical Analysis

Descriptive statistics (mean, median, range) were applied to all numerical data in the study.

## Figures and Tables

**Figure 1:**
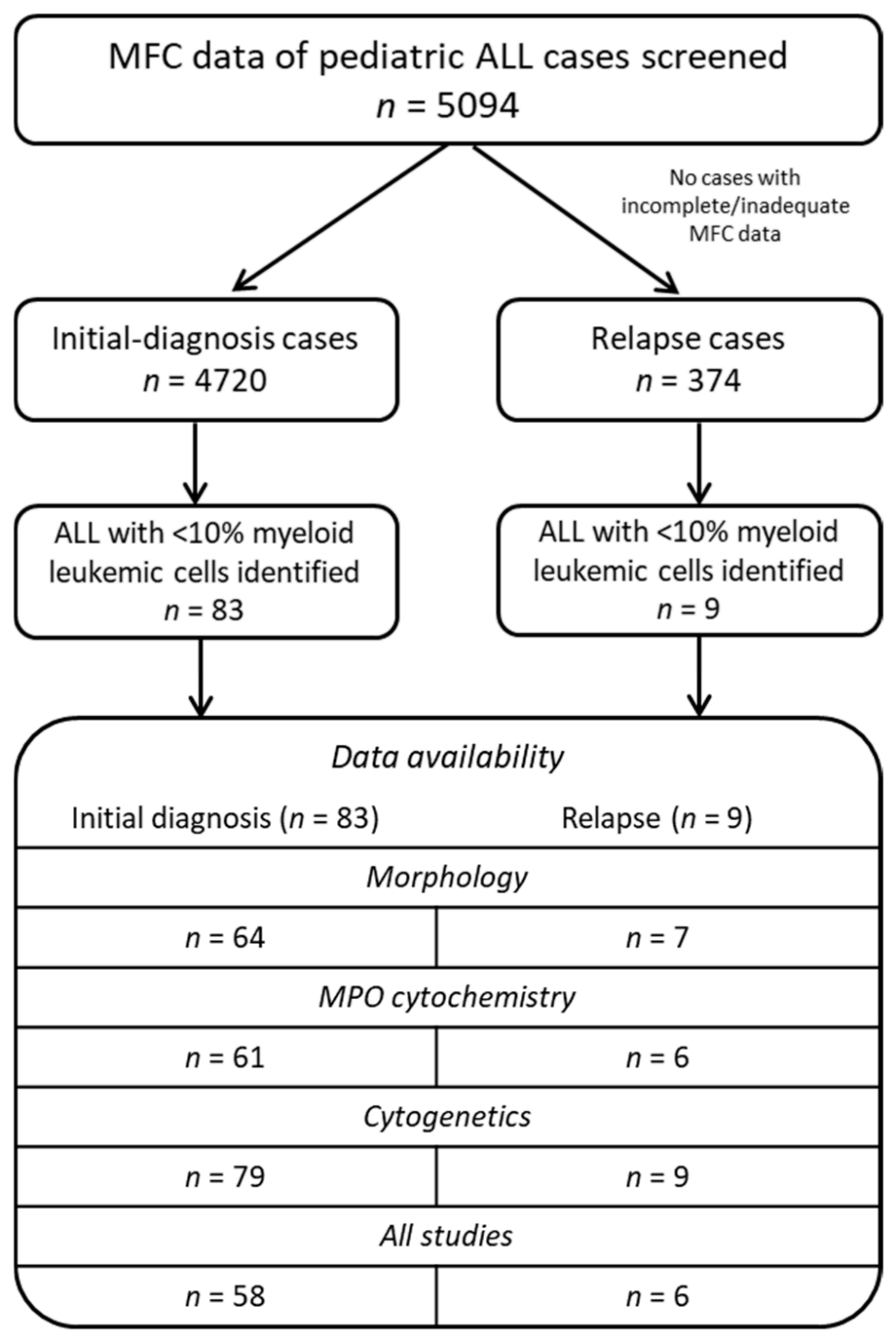
Flow diagram of case selection and the availability of diagnostic laboratory data. ALL—acute lymphoblastic leukemia, MFC—multicolor flow cytometry.

**Figure 2:**
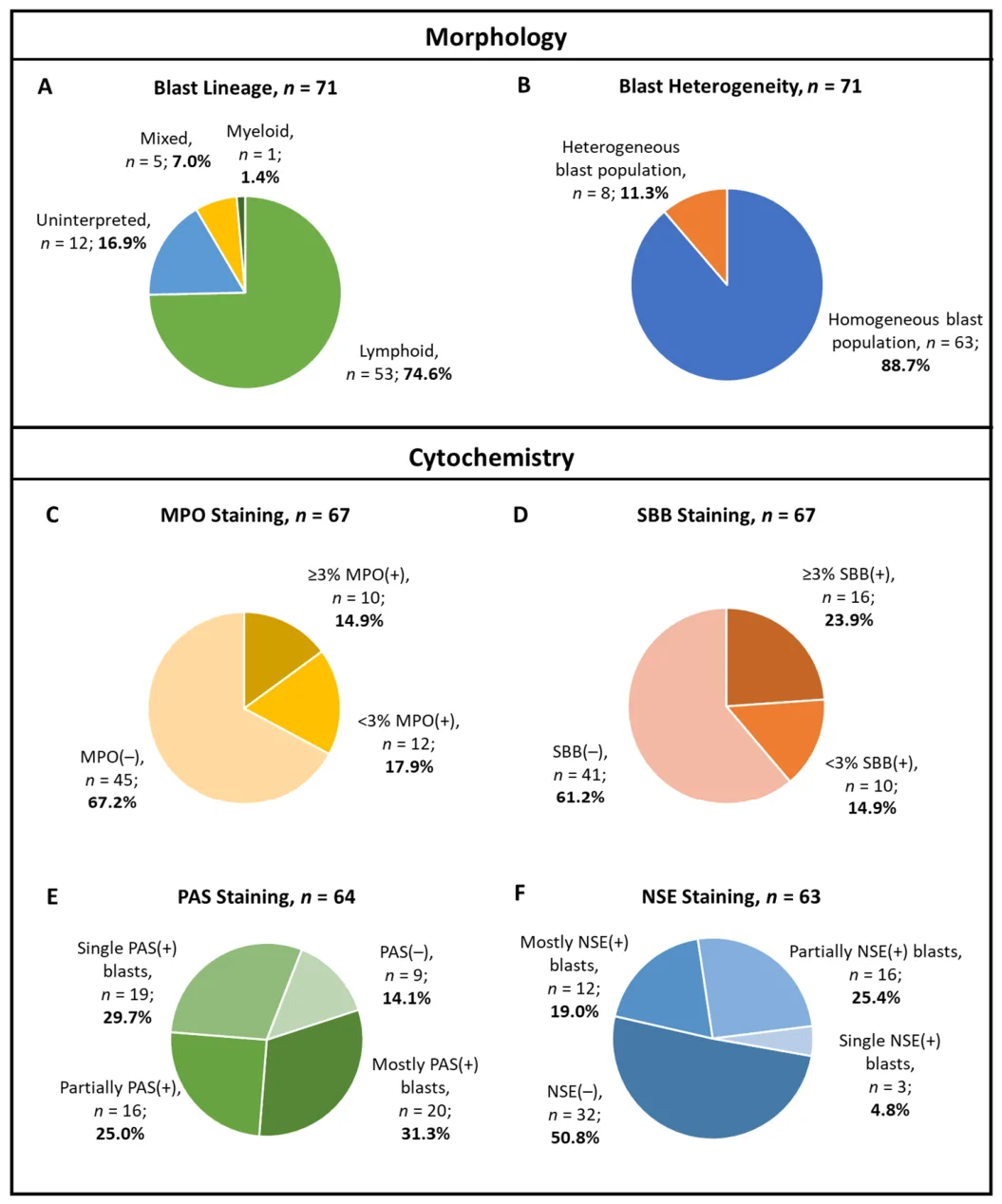
Morphological and cytochemical findings in ALL samples with <10% myeloid leukemic cells: cytological diagnosis distribution (**A**), blast population heterogeneity (**B**), and staining patterns for MPO (**C**), SBB (**D**), PAS (**E**), and NSE (**F**). Percentages may not total 100% because of rounding.

**Figure 3:**
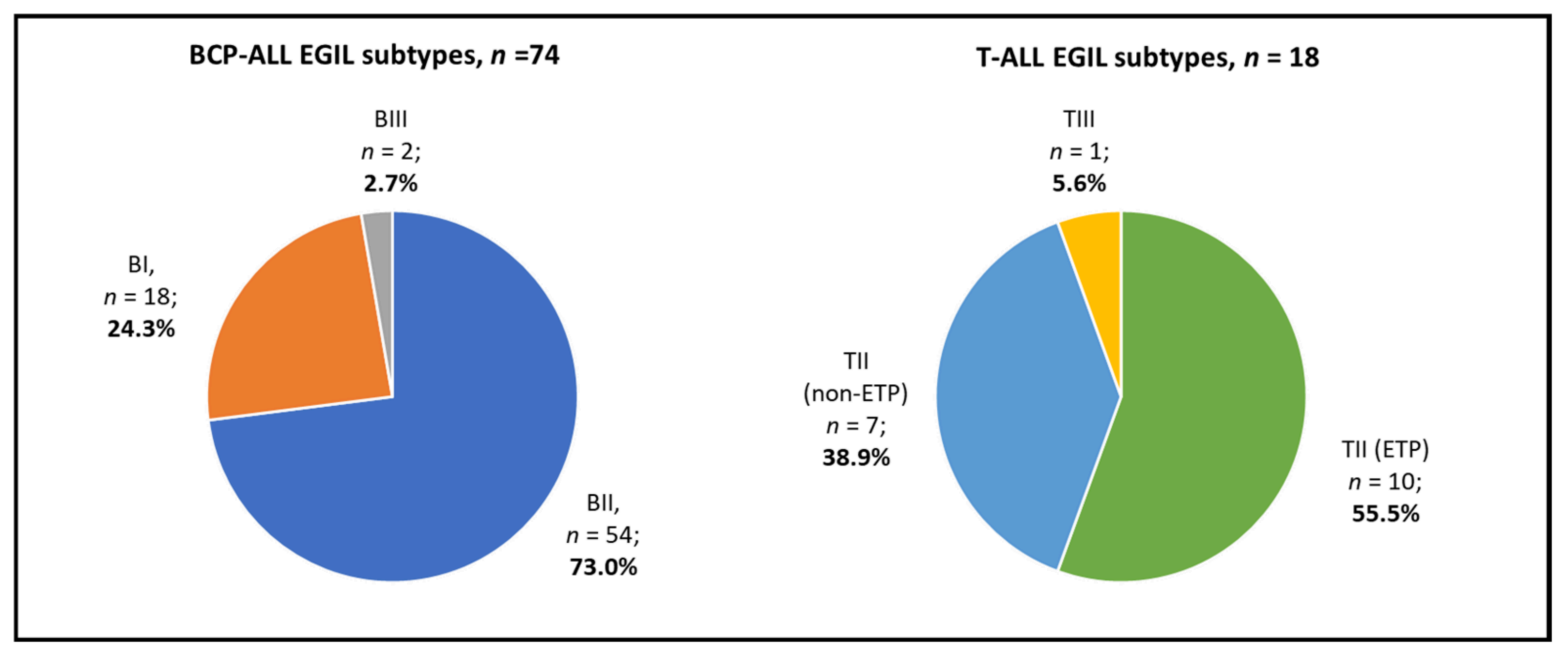
EGIL ALL subtypes revealed in ALL samples with minor myeloid leukemic populations.

**Figure 4:**
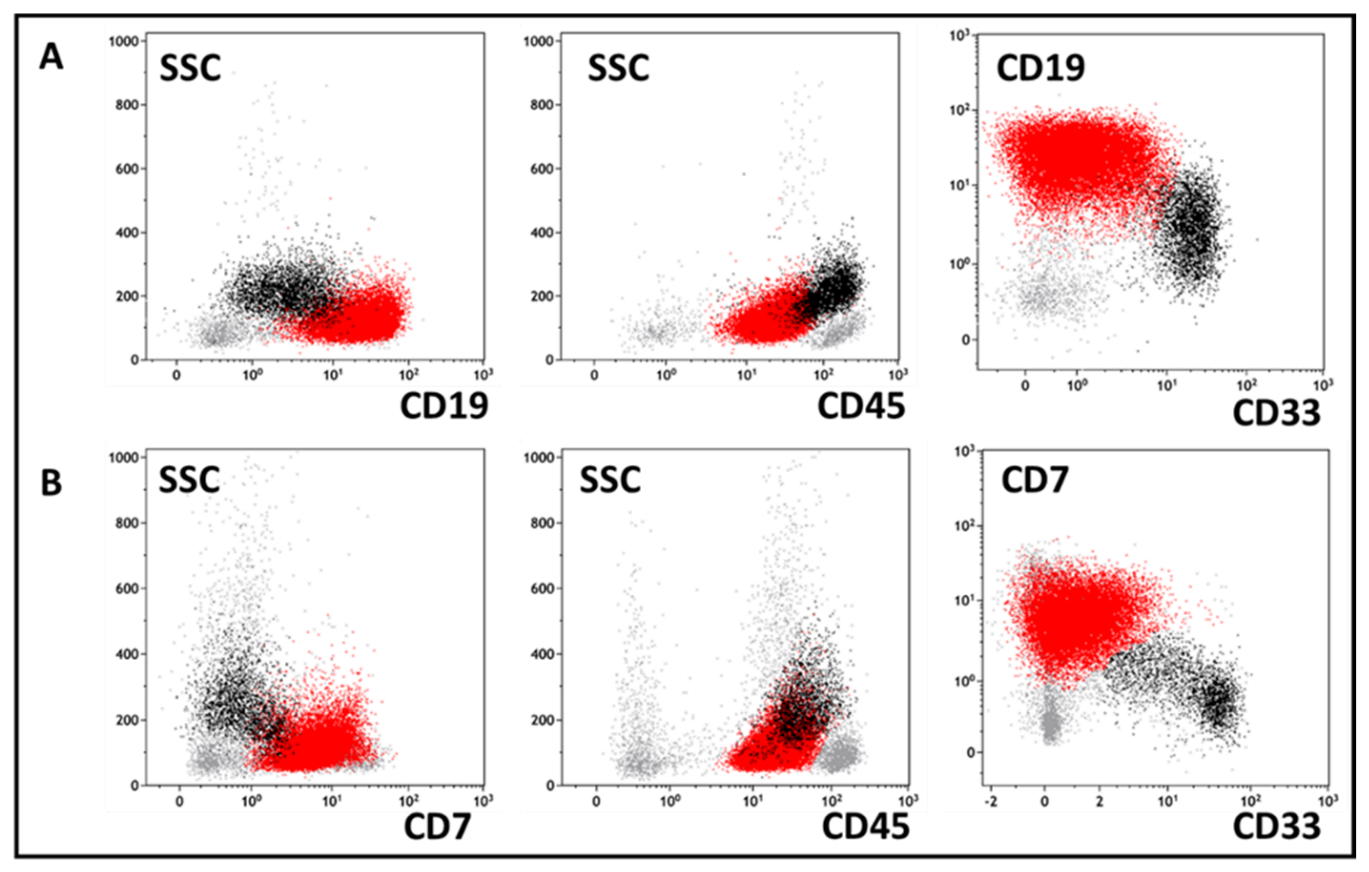
Representative antigen expression patterns of lymphoblasts and myeloid leukemic cells in BCP-ALL (**A**) and T-ALL (**B**). Lymphoblasts are shown in red, myeloid cells in black, and remaining cells in gray.

**Figure 5:**
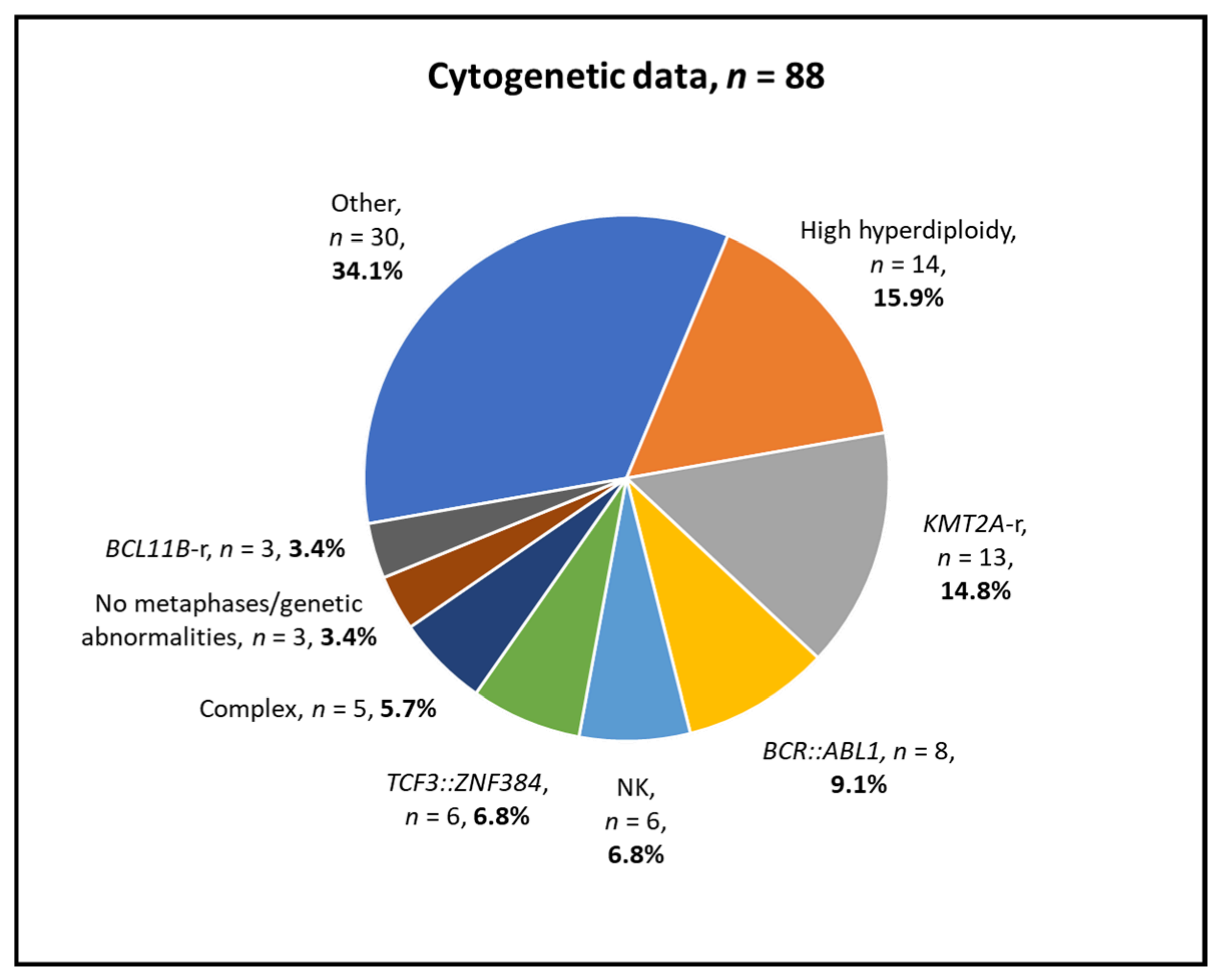
Cytogenetic findings in ALL samples with minor myeloid leukemic populations. NK—normal karyotype.

**Table 1:** Expression of different antigens in myeloid leukemic cells and lymphoblasts in ALL patients. A dash indicates that the corresponding marker was not assessed. i—intracellular staining.

Antigen	BCP-ALL (*n* = 74)				T-ALL (*n* = 18)			
	Myeloid Leukemic Cells		B Lymphoblasts		Myeloid Leukemic Cells		T Lymphoblasts	
	*n* Positive/*n* Analyzed	%	*n* Positive/*n* Analyzed	%	*n* Positive/*n* Analyzed	%	*n* Positive/*n* Analyzed	%
CD2	4/57	7	5/57	9	7/18	39	14/18	78
CD7	0/74	0	5/74	7	13/18	72	18/18	100
CD10	4/74	5	55/74	74	—	—	—	—
CD11c	18/22	82	0/22	0	11/16	69	3/16	19
CD13	34/63	54	25/63	40	13/18	72	10/18	56
CD14	61/74	82	0/74	0	9/18	50	0/18	0
CD15	44/73	60	19/73	26	—	—	—	—
CD19	67/74	91	74/74	100	0/18	0	0/18	0
CD22	13/71	18	65/71	92	—	—	—	—
CD24	20/53	38	37/53	70	—	—	—	—
CD33	70/74	95	27/74	36	18/18	100	12/18	67
CD34	44/74	59	61/74	82	10/18	56	15/18	83
CD38	47/53	89	53/53	100	—	—	—	—
CD45	74/74	100	61/74	82	18/18	100	18/18	100
CD58	47/51	92	51/51	100	—	—	—	—
CD64	65/70	93	0/70	0	14/16	88	1/18	6
iCD79a	2/74	3	74/74	100	0/18	0	4/18	22
CD99	—	—	—	—	14/14	100	14/14	100
CD117	8/74	11	4/74	5	10/18	56	12/18	67
HLA-DR	—	—	—	—	12/16	75	13/16	81
Lysozyme	61/74	82	0/74	0	16/18	89	0/18	0
MPO	23/74	31	0/74	0	15/18	83	0/18	0
NG2	5/74	7	12/74	16	—	—	—	—

**Table 2:** Results of genetic/molecular assessment in cells isolated by flow cell sorting. NK—normal karyotype.

*N*	Immunophenotype	Cytogenetics	Additional Testing
1	79% TII ETP lymphoblasts, 6% myeloid leukemic cells	*BCL11B* rearrangement	*BCL11B* rearrangement in both populations (FISH)
2	65% BII lymphoblasts, 7% myeloid leukemic cells	NK	Identical clonal rearrangements of *IGH* locus (PCR)
3	84% BI lymphoblasts, 5% myeloid leukemic cells	t(4;11)(q21;q23) *KMT2A*::*AFF1*	*KMT2A* rearrangement in both populations (FISH)
4	90% BI lymphoblasts, 7% myeloid leukemic cells	t(11;19)(q23;p13.3) *KMT2A*::*MLLT1*	*KMT2A* rearrangement in both populations (FISH)
5	42% BI lymphoblasts, 7% myeloid leukemic cells	t(12;19)(p13;p13) *TCF3*::*ZNF384*	*ZNF384* rearrangement in both populations (FISH)

**Table 3:** Treatment details of the studied patients. CR—complete remission, LFU—lost to follow-up, LS—lineage switch.

Treatment protocol, *n* = 92	
Newly diagnosed patients, *n* = 83	ALL-MB 2015, *n* = 66
	ALL-IC BFM 2009, *n* = 3
	ALL-BFM 2000, *n* = 1
	ALL-MB-Pilot 2019, *n* = 1
	ALL-2016, *n* = 1
	ALL-Baby 2021, *n* = 4 MLL-Baby, *n* = 3 Mixed/sequential protocols, *n* = 3 Died before therapy initiation, *n* = 1
Relapsed patients, *n* = 9	ALL-MB REZ 2014/2016, *n* = 6
	ALL-BFM REZ 2002/2013, *n* = 2
	Died before therapy initiation, *n* = 1
Refractory disease/subsequent relapse	
Refractory, *n*	8
Relapse, *n*	5
Immunotherapy/HSCT	
Blinatumomab, *n*	25
CAR-T, *n*	6
Inotuzumab, *n*	4
HSCT, *n*	17
LS timepoints	
No subsequent lineage switch, *n*	86
Day 15, *n*	5
At relapse, *n*	1
Outcomes	
Alive in CR, *n*	71
LFU, *n*	10
Death from disease progression, *n*	9
Death from LS, *n*	1
Death from an unspecified cause, *n*	1

**Table 4:** Details of the lineage switch (LS) events in the studied cohort. CR—complete remission, HSCT—hematopoietic stem cell transplantation, i—intracellular staining.

*N*	Sex/Age	Initial Immunophenotype and Genetics	Initial Therapy	LS Timepoint	LS Immunophenotype and Genetics	Post-LS Therapy	Outcome
1	F/1.2 y	84% B lymphoblasts: CD19+iCD79a+CD22+CD34+CD15+CD45+NG2+ 5% myeloid leukemic cells: CD19+CD34+CD45+CD33+CD64+lysozyme+; t(4;11)(q21;q23)*/KMT2A*::*AFF1*	ALL-MB 2015 induction	Day 15	23% myeloid leukemic cells: CD33+CD34+CD45+CD15+CD64+CD11c+lysozyme+; t(4;11)(q21;q23)*/KMT2A*::*AFF1*	ALL-MB 2015	Alive in CR
2	M/15.5 y	95% B lymphoblasts: CD19+iCD79a+CD22+CD34+CD45+NG2+ 2% myeloid leukemic cells: CD19+CD34+CD45+CD33+CD64+CD15+NG2+lysozyme+; t(4;11)(q21;q23)*/KMT2A*::*AFF1*	ALL-MB 2015 induction	Day 15	47% myeloid leukemic cells: CD19−CD34+CD45+CD33+CD15+; t(4;11)(q21;q23)*/KMT2A*::*AFF1*	ALL-MB 2015, HSCT	Alive in CR
3	F/5.0 m	90% B lymphoblasts: CD19+iCD79a+CD45+NG2+ 7% myeloid leukemic cells: CD19−CD14+CD45+CD33+CD64+lysozyme+; t(11;19)(q23;p13.3)*/KMT2A*::*MLLT1*	ALL Baby 2021 induction	Day 15	8% B lymphoblasts: CD19+iCD79a+CD45+ 62% myeloid leukemic cells: CD19+CD14+CD45+CD33+; t(11;19)(q23;p13.3)*/KMT2A*::*MLLT1*	ALL Baby 2021	Alive in CR
4	M/10.0 y	91% B lymphoblasts: CD19+CD10+iCD79a+CD22+CD34+CD45+CD13+CD15+ 3% myeloid leukemic cells: CD19+CD14+CD45+CD33+CD64+lysozyme+; t(4;14)(q35;q32)*/IGH*::*DUX4*	ALL-MB 2015, blinatumomab	10 months after diagnosis	90% myeloid leukemic cells: CD19−CD14−CD45+CD33+CD64+lysozyme+; t(14;4)(q32;q35)*/IGH*::*DUX4*	AML-MRD-2018	Death from LS
5	F/9.6 y	93% B lymphoblasts: CD19+iCD79a+iIgM+CD34+CD45+NG2+ 3% myeloid leukemic cells: CD19+CD34+CD45+CD33+CD64+lysozyme+; t(4;11)(q21;q23)*/KMT2A*::*AFF1*	ALL-MB 2015 induction	Day 15	0.841% B lymphoblasts: CD19+CD34+CD45+ 4.584% myeloid leukemic cells: CD19−CD34+CD45+CD33+; t(4;11)(q21;q23)*/KMT2A*::*AFF1*	AML-BFM 2020	Alive in CR
6	M/14.7 y	95% T lymphoblasts (ETP): iCD3+CD7+CD2+CD34+CD117+CD13+CD45+ 5% myeloid leukemic cells: CD34+CD45+CD33+CD117+CD13+CD15+CD64+MPO+; 47,XY,+4,der(11)?[1]/ 46,XY[7]	ALL-MB 2015 induction	Day 15	42% myeloid leukemic cells: iCD3−CD7+CD2+CD34+CD117+CD13+CD45+; Genetic studies were not performed	ALL-MB 2015, HSCT	Death from unspecified cause

## Data Availability

The original contributions presented in the study are included in the article; further inquiries can be directed to the corresponding author.

## Abbreviations

The following abbreviations are used in this manuscript:

ALL	acute lymphoblastic leukemia
ALL-BFM	Acute Lymphoblastic Leukemia–Berlin–Frankfurt–Münster protocol
ALL-MB	Acute Lymphoblastic Leukemia–Moscow–Berlin protocol
AML	acute myeloid leukemia
BCP-ALL	B-cell precursor acute lymphoblastic leukemia
BM	bone marrow
CAR-T	chimeric antigen receptor T-cell therapy
CD	cluster of differentiation
CR	complete remission
EGIL	European Group for the Immunological Characterization of Leukemias
EOI	end of induction
ETP-ALL	early T-cell precursor acute lymphoblastic leukemia
FAB	French–American–British classification
FISH	fluorescence in situ hybridization
HSCT	hematopoietic stem cell transplantation
i	intracellular
iAMP21	intrachromosomal amplification of chromosome 21
IG	immunoglobulin
ISCN	International System for Human Cytogenomic Nomenclature
LFU	lost to follow-up
LS	lineage switch
MFC	multicolor flow cytometry
M-FISH	multicolor fluorescence in situ hybridization
MPAL	mixed-phenotype acute leukemia
MPO	myeloperoxidase
MRD	measurable residual disease
NK	normal karyotype
NOS	not otherwise specified
NSE	α-naphthyl acetate esterase
PAS	periodic acid–Schiff
RNAseq	RNA sequencing
RT-PCR	reverse transcription polymerase chain reaction
SBB	Sudan Black B
T-ALL	T-cell acute lymphoblastic leukemia
TCR	T-cell receptor
WGS	whole-genome sequencing
WHO	World Health Organization

## Appendix A

**Table A1:** Summary of karyotypic, FISH and molecular findings in ALL samples with minor myeloid leukemic populations (*n* = 92) and corresponding categories according to the International Consensus Classification (ICC).

*N*	Sex, Age	ALL Subtype	Genetics (Summary)	ICC Category
1	F, 10 y	BII	45,XX,-7[9]/46,XX[1]; FISH: Monosomy 7	B-ALL without recurrent genetic abnormalities
2	F, 16 y	TII ETP	46,XX[20]; FISH: No risk-stratifying aberrations; Sanger sequencing: *FLT3*-ITD	Early T-cell precursor ALL, NOS
3	M, 12 y	BI	46,XY[11]; FISH: No risk-stratifying aberrations; WGS: *PAX5* P80R	B-ALL with *PAX5* P80R
4	M, 8 y	BII	50,XY,+X,+8,+20,+22[cp2]; FISH: No risk-stratifying aberrations	B-ALL hyperdiploid
5	M, 10 y	BII	50–51,XY,+X,der(2),+6,+6,der(9)?,-12,+14,+21,+21,+mar1–3[cp7]; FISH: del(17p)	B-ALL hyperdiploid
6	M, 15 y	TII non-ETP	47,XY,der(9),der(9_),+der(9),del(11q),der(12)[4]; FISH: No risk-stratifying aberrations; WGS: t(14;16)(q32.2;q24.1)/*IGH*::*BCL11B*	T-ALL, NOS
7	M, 6 y	BII	54,XY,+X,+Y,+4,+6,+der(10),+14,der(16)?,der(19),+21,+21[2]; FISH: No risk-stratifying aberrations	B-ALL hyperdiploid
8	F, 4 y	BII	57,XX,+X,der(1),+4,+7,+8,+10,+13,+17,+18,+21,+21,+mar[2]; FISH: No risk-stratifying aberrations	B-ALL hyperdiploid
9	M, 16 y	TII ETP	47,XY,+5?[3]; FISH: *RUNX1* rearrangement; Multiplex anchor PCR: inv(21)(q22.12q22.3)/*UMODL1*::*RUNX1*	Early T-cell precursor ALL, NOS
10	F, 7 y	BI	46,XX[10]; FISH: No risk-stratifying aberrations	B-ALL without recurrent genetic abnormalities
11	F, 5 y	BII	56,XX[1]; FISH: No risk-stratifying aberrations	B-ALL hyperdiploid
12	F, 3 y	BII	No metaphases; FISH: Trisomy 4, 10, 17	B-ALL hyperdiploid
13	F, 4 y	BII	del(12p) FISH: No risk-stratifying aberrations	B-ALL without recurrent genetic abnormalities
14	F, 13 y	BII	44,XX,-9,der(12)t(12;17)(p12;q12),-17[2]; FISH: del(17p); Sanger sequencing: Somatic *TP53* mutation	B-ALL without recurrent genetic abnormalities
15	M, 6 y	BII	No metaphases; FISH: t(9;22)(q34;q11)/*BCR*::*ABL1*; RT-PCR: *BCR*::*ABL1* p190	B-ALL with t(9;22)(q34;q11.2)/*BCR*::*ABL1*
16	M, 9 y	TII ETP	46,XY[2]/46,XY,der(11)?[1], low-quality metaphases; FISH: No risk-stratifying aberrations; Sanger sequencing: *FLT3*-ITD	Early T-cell precursor ALL, NOS
17	M, 10 y	BII	46,XY[9]; FISH: t(9;22)(q34;q11)/*BCR*::*ABL1*; RT-PCR: *BCR*::*ABL1* p190	B-ALL with t(9;22)(q34;q11.2)/*BCR*::*ABL1*
18	F, 7 y	TII ETP	46,XX[6]; FISH: No risk-stratifying aberrations; WGS: t(6;14)(q25.3;q32.2)/*BCL11B*::*ARID1B*	Early T-cell precursor ALL, *BCL11B*-activated
19	F, 15 d	BI	46,XX,t(4;11)(q21;q23)[10]; FISH: t(4;11)(q21;q23)/*KMT2A*::*AFF1*	B-ALL with *KMT2A* rearrangement
20	M, 18 y	BII	46,XY[5]; FISH: No risk-stratifying aberrations	B-ALL without recurrent genetic abnormalities
21	F, 4 m	BI	46,XX,t(4;11)(q21;q23)[29]/46,XX[1]; FISH: t(4;11)(q21;q23)/*KMT2A*::*AFF1*	B-ALL with *KMT2A* rearrangement
22	M, 14 y	BII	46,XY[11]; FISH: No risk-stratifying aberrations; WGS: *PAX5* P80R	B-ALL with *PAX5* P80R
23	F, 1 y	BI	46,XX,t(4;11)(q21;q23)[2]; FISH: t(4;11)(q21;q23)/*KMT2A*::*AFF1*	B-ALL with *KMT2A* rearrangement
24	M, 17 y	BII	46,XY,del(9p)[1]/47,XY,+8,del(9p)[5]; FISH: No risk-stratifying aberrations;	B-ALL without recurrent genetic abnormalities
25	M, 16 y	BI	46,XY[10]; FISH: No risk-stratifying aberrations;	B-ALL without recurrent genetic abnormalities
26	M, 15 y	BI	46,XY,t(4;11)(q21;q23)[9]/46,XY[3]; FISH: t(4;11)(q21;q23)/*KMT2A*::*AFF1*	B-ALL with *KMT2A* rearrangement
27	M, 15 y	BII	48–54,XY,+4,+6,+10,+12,+16,+18,+20,+21,+22[cp5]/46,XY[15]; FISH: No risk-stratifying aberrations;	B-ALL hyperdiploid
28	F, 5 d	BI	46,XX,t(4;11)(q21;q23)[5]; FISH: t(4;11)(q21;q23)/*KMT2A*::*AFF1*	B-ALL with *KMT2A* rearrangement
29	M, 12 y	BII	46,XY,t(9;22)(q34;q11),der(9)[3]; FISH: t(9;22)(q34;q11)/*BCR*::*ABL1*; RT-PCR: *BCR*::*ABL1* p190	B-ALL with t(9;22)(q34;q11.2)/*BCR*::*ABL1*
30	M, 12 y	TII ETP	47,XY,+4[17]/46,XY[3]; FISH: No risk-stratifying aberrations	Early T-cell precursor ALL, NOS
31	M, 7 m	BI	46,XY,t(4;11)(q21;q23)[5]/46,XY[1]; FISH: t(4;11)(q21;q23)/*KMT2A*::*AFF1*	B-ALL with *KMT2A* rearrangement
32	F, 5 m	BI	46,XX,t(11;19)(q23;p13.3)[20]; FISH: t(11;19)(q23;p13.3)/*KMT2A*::*MLLT1*	B-ALL with *KMT2A* rearrangement
33	F, 9 y	BII	45,XX,-7,del(9)(p21)[20]; FISH: Monosomy 7	B-ALL without recurrent genetic abnormalities
34	M, 9 y	TII non-ETP	45,XY,der(1),der(7),der(12),-15[9]/46,XY,der(1),der(7)[6]/46,XY[2]; FISH: No risk-stratifying aberrations;	T-ALL, NOS
35	M, 11 y	BII	52,XY,+X,+14,+18,+21,+21,+der[3]; FISH: No risk-stratifying aberrations	B-ALL hyperdiploid
36	F, 13 y	BII	46,XX,der(22)t(1;22)(q25;q13)[12]/46,XX,der(4),der(6),del(11)(q14),-18,+mar[1]/46,XX[5]; FISH: No risk-stratifying aberrations	B-ALL without recurrent genetic abnormalities
37	F, 7 y	BI	No metaphases; FISH: t(12;19)(p13;p13)/*TCF3*::*ZNF384*	B-ALL with *ZNF384* rearrangement
38	M, 13 y	TII ETP	46,XY,der(2),del(12p)[4]/46,XY[6]; FISH: No risk-stratifying aberrations	Early T-cell precursor ALL, NOS
39	F, 17 y	BII	45–48,XX,t(1;11)(p32;q23),+der(1)t(1;11),+6,del(9p),del(14)?der(19),der(21)?[cp3]; RNAseq: t(1;11)(p34.2;q23)/*KMT2A*::*CTPS1*	B-ALL with *KMT2A* rearrangement
40	F, 2 y	BII	46,XX,del(9q)[1]/46,XX[6]; FISH: del 3′-*TCF3*; WGS: t(12;19)(p13;p13)/*TCF3*::*ZNF384*	B-ALL with *ZNF384* rearrangement
41	F, 13 y	BII	45–47,XX,+der(6)?,der(7),del(9p),der(11)?,i(17)(q10)[cp4]; FISH: del(17p)	B-ALL without recurrent genetic abnormalities
42	M, 4 y	TII ETP	46,XY,t(8;12)(q13;p13),del(9p),der(21)[7]/46,XY[2]; FISH: *RUNX1* rearrangement, del 3′-*ETV6*, del 5′-*PAX5*	Early T-cell precursor ALL, NOS
43	M, 14 y	TII	47–48,XY,+X,+Y,der(1),der(5),+8,del(9p),der(12)add(p13)[cp3]; FISH: Trisomy 8	T-ALL, NOS
44	M, 7 y	BII	46,XY[1]; FISH: *PDGFRB* rearrangement	B-ALL *BCR*::*ABL1*-like*,* ABL-1 class rearranged
45	F, 12 y	BII	46,XX[5]; FISH: t(12;19)(p13;p13)/*TCF3*::*ZNF384*	B-ALL with *ZNF384* rearrangement
46	F, 7 y	TII	No metaphases; FISH: *TLX3* rearrangement	T-ALL, NOS
47	F, 5 y	BII	25,X-X,-2,-3,-4,-5,-6,-7,-8,-9,-10,-11,-12,-13,-15,-16,-17,-19,-20,-21,-22[cp3]; FISH: No aberrations	B-ALL near haploid
48	M, 10 y	BII	No metaphases; FISH: del 3′-*IGH* WGS: t(14;4)(q32;q35)/*IGH*::*DUX4*	B-ALL with *DUX4* rearrangement
49	F, 1 y	BIII	46,XX,t(2;9)(p13;p13),del(13q),del(20q)[4]; FISH: *PAX5* rearrangement WGS: t(9;2)(p13;p11.2)/*PAX5*::*IGK*	B-ALL with other recurrent genetic abnormalities
50	F, 9 y	BII	47,XX,+X[7]/46,XX[3]; FISH: t(X;14)(p22;q32)/*CRLF2*::*IGH*	B-ALL with other recurrent genetic abnormalities
51	F, 11 y	BII	46,XX[3], low-quality metaphases; FISH: No risk-stratifying aberrations	B-ALL without recurrent genetic abnormalities
52	M, 16 y	BII	46,XY,der(8)add(p23),der(9)[5]/46,XY[1]; FISH: del *PAX5*, del *JAK2*	B-ALL without recurrent genetic abnormalities
53	M, 12 y	BII	46,XY,t(9;22)(q34;q11)[2]; FISH: t(9;22)(q34;q11)/*BCR*::*ABL1*; RT-PCR: *BCR*::*ABL1* p190	B-ALL with t(9;22)(q34;q11.2)/*BCR*::*ABL1*
54	M, 17 y	BII	No cytogenetic/molecular data	no cytogenetic/molecular data
55	M, 8 y	TII ETP	46,XY[5]; FISH: *CMYC* rearrangement	Early T-cell precursor ALL, NOS
56	M, 6 y	TII	No cytogenetic/molecular data	no cytogenetic/molecular data
57	F, 3 y	BII	62[2]; FISH: Trisomy 4, 10, 17	B-ALL hyperdiploid
58	M, 3 y	BII	46,XY[16]; FISH: t(12;19)(p13;p13)/*TCF3*::*ZNF384*	B-ALL with *ZNF384* rearrangement
59	F, 2 y	TIII	46,XX,t(9;22)[6]/46,XX,der(7)inv(7)?,t(9;22)[4]; FISH: t(9;22)(q34;q11)/*BCR*::*ABL1*; RT-PCR: *BCR*::*ABL1* p210	T-ALL, NOS
60	M, 15 y	BII	No cytogenetic/molecular data	no cytogenetic/molecular data
61	M, 3 y	BII	Trisomy 11, tetrasomy 21, del(19p) FISH: No risk-stratifying aberrations	B-ALL without recurrent genetic abnormalities
62	M, 2 y	BII	No metaphases; FISH: del 3′-*ETV6*; WGS: *PRH1*::*ETV6*, +6,+10,+10,+18,+21,+21,+22,+X,-Y	B-ALL with other recurrent genetic abnormalities
63	M, 5 y	BII	Trisomy 21; FISH: No risk-stratifying aberrations	B-ALL without recurrent genetic abnormalities
64	M, 14 y	BI	FISH: t(4;11)(q21;q23)/*KMT2A*::*AFF1*	B-ALL with *KMT2A* rearrangement
65	M, 12 y	BII	No metaphases; FISH: No risk-stratifying aberrations	B-ALL without recurrent genetic abnormalities
66	F, 7 y	BII	47,XX,+8[5]; FISH: No risk-stratifying aberrations	B-ALL without recurrent genetic abnormalities
67	F, 4 y	BII	No metaphases; FISH: Trisomy 4, 10, 17	B-ALL hyperdiploid
68	M, 4 y	BII	46,XY,t(9;22)(q34;q11)[4]/46,XY,t(9;22)(q34;q11),der(16)add(16q)?t(1;16)[3]; FISH: t(9;22)(q34;q11)/*BCR*::*ABL1*; RT-PCR: *BCR*::*ABL1* p190	B-ALL with t(9;22)(q34;q11.2)/*BCR*::*ABL1*
69	M, 3 y	BII	Tetrasomy 21; FISH: No risk-stratifying aberrations	B-ALL without recurrent genetic abnormalities
70	F, 9 y	BII	55,XX[2]; FISH: No risk-stratifying aberrations	B-ALL hyperdiploid
71	M, 16 y	BII	Trisomy 21; FISH: No risk-stratifying aberrations	B-ALL without recurrent genetic abnormalities
72	M, 17 y	BII	46,XY,der(9),der(20)[15]/46,XY,der(9),der(9),der(20)[4]/46,XY[1]; FISH: No risk-stratifying aberrations	B-ALL without recurrent genetic abnormalities
73	M, 5 y	BII	47,XY,+X,der(21)(iAMP?)[3]; FISH: iAMP21, del(X)(p22;p22)/*CRFL2*::*P2RY8*	B-ALL with iAMP21
74	M, 3 y	BI	45,XY,-18[3]/46,XY[16]; FISH: t(4;11)(q21;q23)/*KMT2A*::*AFF1*	B-ALL with *KMT2A* rearrangement
75	F, 17 y	BII	No metaphases; FISH: No risk-stratifying aberrations	B-ALL without recurrent genetic abnormalities
76	F, 4 y	BII	55–56,XX,+X,+3,+6,+10,+13,+14,+14,+15,+18,+18,+21,+21[cp4]; FISH: No risk-stratifying aberrations	B-ALL hyperdiploid
77	M, 7 y	TII non-ETP	46,XY,der(3),der(6),der(8),der(9),der(10),der(11),der(12)t(12;14)(p12;q21),der(14)t(12;14)(p12;q21)[cp10]; FISH: No risk-stratifying aberrations	T-ALL, NOS
78	F, 9 y	BIII	46,XX,t(4;11)(q21;q23)[3]; FISH: t(4;11)(q21;q23)/*KMT2A*::*AFF1*	B-ALL with *KMT2A* rearrangement
79	M, 12 y	BII	46,XY,-7,del(2)(q32),add(5)(p15),der(7),der(10),der(12),+mar[15]; FISH: del 5′-*IGH* *IKZF1* rearrangement	B-ALL with other recurrent genetic abnormalities
80	F, 3 y	BI	47,XX,+mar[3]/46,XX[2]; FISH: t(12;19)(p13;p13)/*TCF3*::*ZNF384*	B-ALL with *ZNF384* rearrangement
81	M, 14 y	TII ETP	47,XY,+4,der(11)?[1]/46,XY[7]; FISH: No risk-stratifying aberrations	Early T-cell precursor ALL, NOS
82	M, 16 y	BII	No metaphases; FISH: t(9;22)(q34;q11)/*BCR*::*ABL1*; RT-PCR: *BCR*::*ABL1* p210	B-ALL with t(9;22)(q34;q11.2)/*BCR*::*ABL1*
83	M, 3 y	BII	46,XY,t(9;22)(q34;q11),i(12)(q10)[3]; FISH: t(9;22)(q34;q11)/*BCR*::*ABL1*; RT-PCR: *BCR*::*ABL1* p190	B-ALL with t(9;22)(q34;q11.2)/*BCR*::*ABL1*
84	M, 15 y	TII	51,der(7)+8?,+8?,+mar1,+mar2,+mar3[1]; FISH: No risk-stratifying aberrations; WGS: *PAX5* mutation	T-ALL, NOS
85	M, 19 d	BI	46,XY,t(4;11)(q21;q23)[5]; FISH: t(4;11)(q21;q23)/*KMT2A*::*AFF1*	B-ALL with *KMT2A* rearrangement
86	F, 3 m	BI	46,XX,t(12;17)(p13;q11),der(19),del(20q)[5]; FISH: t(12;19)(p13;p13)/*TCF3*::*ZNF384*	B-ALL with *ZNF384* rearrangement
87	F, 4 y	BII	54,XX,+X,der(10)dup(1q),+4,+10,+14,der(16),+17,+18,+21,+21[2]; FISH: No risk-stratifying aberrations	B-ALL hyperdiploid
88	M, 8 y	BII	No cytogenetic/molecular data	no cytogenetic/molecular data
89	M, 14 y	TII ETP	46,XY,t(6;7)(q27;p12),der(14)add(14q32)[4]; M-FISH: *BCL11B* rearrangement in t(6;7;14)(q27;p12;q32)	Early T-cell precursor ALL, *BCL11B*-activated
90	M, 2 y	BII	No metaphases; FISH: No risk-stratifying aberrations	B-ALL without recurrent genetic abnormalities
91	F, 8 y	BI	46,XX,t(4;11)(q21;q23)[3]; FISH: t(4;11)(q21;q23)/*KMT2A*::*AFF1*	B-ALL with *KMT2A* rearrangement
92	F, 4 y	BII	58–59,XX,+X,+2,+6,+10?,+13,+14,+18,+18,+21,+21,+mar[cp3]; FISH: No risk-stratifying aberrations	B-ALL hyperdiploid
